# Ultrasound-Guided Peripheral Intravenous Access Using Different Vein-Selection Strategies in Difficult Emergency Department Patients: A Systematic Review

**DOI:** 10.7759/cureus.117797

**Published:** 2026-10-12

**Authors:** Alaa Albadwy, Ammar Alemam Deiab Alnour, Nada Fathi Mohammed Ahmed Hamid, Maryam Abdulrahman Osman Derar, Sohail Ibrahim Alhussein Alamass, Mohamed Samir Yasin Ibrahim, Yasin Samir Yasin Ibrahim, Anas Salaheldin Ahmed Khalil, Hanady ME M Osman

**Affiliations:** 1 Emergency Medicine, United Lincolnshire Teaching Hospitals NHS Trust, Lincoln, GBR; 2 Medicine and Surgery, Almughtaribeen University, Khartoum, SDN; 3 Internal Medicine, Dr. Sulaiman Al Habib Medical Group, Riyadh, SAU; 4 Emergency Medicine, Dr. Suliman Al Habib Hospital, Riyadh, SAU; 5 Emergency Medicine, Quriyat Hospital, Ministry of Health, Quriyat, OMN; 6 Pathophysiology, School of Medicine, St. George’s University, St. George, GRD; 7 Emergency Medicine, Dr. Sulaiman Al Habib Medical Group, Riyadh, SAU; 8 Emergency Medicine, Kettering General Hospital NHS Foundation Trust, Kettering, GBR; 9 Quality Improvement and Patient Safety, Najran Armed Forces Hospital, Ministry of Defense Health Services, Najran, SAU

**Keywords:** catheter dwell time, difficult intravenous access, emergency department, peripheral intravenous catheter, ultrasound-guided intravenous access, vascular ultrasound, vein selection

## Abstract

Ultrasound guidance has become the reference approach to peripheral intravenous cannulation in emergency department (ED) patients with difficult intravenous access (DIVA), yet the single decision that ultrasound uniquely enables - which vein to choose - remains poorly codified. This review synthesises primary evidence on how vein-selection strategies influence cannulation success and catheter longevity.

Four databases were searched from inception to 30 June 2026 for original studies of adult ED patients with DIVA in whom a target-vein selection variable (anatomical site, sonographic depth, sonographic diameter, or catheter-to-vein geometry) was related to an access outcome. Study selection, extraction and risk-of-bias assessment using Risk Of Bias In Non-randomised Studies of Interventions (ROBINS-I) were performed in duplicate. Findings were synthesised narratively; meta-analysis was not appropriate.

Ten studies (1,952 patients or cannulation encounters) met the criteria. Larger vessel diameter predicted insertion success, but no statistically significant association with longevity was detected in the single study that examined it. Increasing vessel depth was consistently associated with poorer catheter survival in the three cohorts that examined longevity, with a threshold near 1.2 cm, and limited insertion success beyond approximately 1.5-1.6 cm in two further studies. Deep proximal upper-arm veins delivered the highest first-pass success but the poorest durability, while forearm and antecubital targets showed the reverse. Three of four studies found that maximising the intraluminal catheter length - a direct consequence of vein choice - protected against premature failure. Overall risk of bias was low in four studies, moderate in two and serious in four.

Vein selection appears to be a modifiable determinant of both obtaining and retaining peripheral access in DIVA patients, and the optimal target differs according to whether the immediate priority is cannulation or dwell time. A prospectively validated, sonographically anchored selection rule has yet to be tested, and the thresholds identified here, which derive from separate cohorts, should be regarded as an evidence-informed approach pending prospective validation rather than a clinical decision rule.

## Introduction and background

Peripheral intravenous cannulation is the most frequently performed invasive procedure in emergency medicine, and it fails often enough to constitute a distinct clinical problem in its own right. Between one in 10 and one in three emergency department (ED) patients meet criteria for difficult intravenous access (DIVA), with obesity, chronic illness, injection drug use, dialysis dependence, oedema, hypovolaemia and repeated prior cannulation all contributing to venous depletion [1]. Prospectively derived risk-factor analyses in adult ED cohorts have confirmed that non-visible and non-palpable veins, a patient-reported history of difficult access and specific comorbidity clusters identify these patients before the first needle is advanced [2]. Despite the frequency of the problem, terminology remained inconsistent for decades; a systematic review of the published definitions concluded that DIVA is best operationalised as two or more failed attempts using traditional techniques, physical examination findings suggestive of difficult access, or a documented history of the same [3]. The clinical consequences of the condition are cumulative rather than dramatic: repeated venipuncture, analgesic and antimicrobial delays, prolonged emergency stays, escalation to more invasive access and an avoidable erosion of the patient's remaining venous capital.

Ultrasound guidance transformed the management of these patients. The pivotal randomised comparison of ultrasonography-guided cannulation with traditional approaches in DIVA patients demonstrated higher success, fewer percutaneous punctures, shorter time to access and improved patient satisfaction [4], and the accumulated randomised evidence has since been synthesised in a meta-analysis showing a robust improvement in first-attempt success for difficult peripheral catheterisation [5]. At a service level, the effect extends beyond the individual cannula: implementation of a departmental ultrasound-guided peripheral access programme was associated with a marked reduction in central venous catheter use in non-critically ill ED patients, displacing a procedure that carries mechanical, infectious and thrombotic risk [6]. Ultrasound guidance is therefore no longer a rescue manoeuvre but the expected standard of care for the DIVA patient in most well-resourced emergency systems.

What ultrasound actually changes, however, is not simply the accuracy of needle advancement but the range of veins the clinician can consider. Before insonation, the operator is restricted to vessels that can be seen or palpated; afterwards, the entire upper-limb venous tree, including deep brachial and basilic vessels several centimetres below the skin, becomes a candidate target, each with a different calibre, depth, course and trajectory length. Evidence-based practice summaries have long recognised that target-vessel characteristics matter, recommending attention to vein size, depth and site alongside probe orientation and catheter length [7]. The link between vein choice and catheter durability is largely geometric. For a catheter of fixed length inserted at a given angle, every additional millimetre of tissue between the skin and the vein consumes catheter that would otherwise lie inside the vessel; a deeper vein therefore leaves a shorter intraluminal segment, which is more easily pulled out of the lumen by limb movement, oedema or dressing traction, producing dislodgement or infiltration. The length of catheter residing within the vein is thus determined jointly by the depth of the chosen vein and the length of the device. Yet these recommendations are drawn from heterogeneous single-centre studies that were designed to answer different questions, employed different outcome definitions, and have never been formally collated. The consequence is that the one decision unique to ultrasound-guided access - selecting which vein to cannulate - is still made largely by operator preference. This systematic review was therefore conducted to identify, appraise and synthesise the primary evidence on how different vein-selection strategies affect cannulation success, catheter survival and complications in adult ED patients with DIVA.

## Review

Materials and methods

Protocol and Reporting

The review was designed and reported in accordance with the Preferred Reporting Items for Systematic Reviews and Meta-Analyses (PRISMA) 2020 statement, and the 27-item PRISMA checklist was completed before submission [8]. A structured protocol specifying the review question, eligibility criteria, search strategy, outcomes and analysis plan was written before the searches were executed; the review was not prospectively registered, which is acknowledged as a limitation.

Search Strategy

PubMed/MEDLINE, Embase (Elsevier), Scopus and Web of Science were searched from database inception to 30 June 2026. The strategy combined controlled vocabulary and free-text terms across four concept blocks joined with the Boolean operator AND, with terms within each block joined by OR. Block 1 (technique) covered ultrasound, sonography and point-of-care ultrasound; block 2 (procedure) covered peripheral intravenous and peripheral venous cannulation and catheterisation; block 3 (population and setting) covered difficult intravenous, venous and vascular access and the emergency department; and block 4 (target-vein descriptors) covered vein depth, vessel depth, vein diameter, vessel calibre, vein width, basilic, brachial, cephalic, forearm, antecubital, upper arm, catheter-to-vein ratio, catheter in vein, target vein and insertion site. The complete search string executed in each database, together with the number of records retrieved from each, is provided in Appendix 1. No date, geographic or publication-status restriction was applied other than the requirement for peer-reviewed original research in English. Reference lists of all included articles and of relevant narrative and systematic reviews were hand-searched, and forward citation tracking was performed in Scopus and Google Scholar.

Eligibility Criteria

Eligibility was framed using the Population, Intervention, Comparison, Outcomes and Study (PICOS) structure and is summarised in Table 1. Studies were eligible if they enrolled adults with DIVA in an ED setting, evaluated ultrasound-guided peripheral cannulation in which at least one target-vein selection variable was specified or measured, compared that variable against an alternative selection strategy or stratum, and reported at least one clinical access outcome. Studies whose only exposure variable was the catheter device or its manufactured length, with no descriptor of the vein selected, were excluded because the exposure of interest in such designs is the device rather than the vein. Studies in which a named target vein was pre-specified for all participants and paired with a particular catheter, such as a deep brachial or basilic target cannulated with a long catheter, were eligible because a vein descriptor defined the exposure; the catheter was recorded as a co-intervention. Single-arm series of a pre-specified target-vein strategy were eligible only when their results could be interpreted against an external reference (contemporaneous standard practice, defined under Risk-of-bias assessment); such a reference is not a concurrent comparison group, and these studies contribute descriptive rather than comparative estimates.

**Table 1:** Eligibility criteria according to the PICOS framework PICOS: Population, Intervention, Comparison, Outcomes and Study

PICOS element	Inclusion criteria	Exclusion criteria
Population	Adults ≥18 years in an emergency department (ED) with difficult intravenous access, defined as ≥2 failed landmark attempts, no visible or palpable veins, or a documented history of difficult access	Age <18 years; cohorts recruited wholly outside the ED; studies in which difficult-access status was not defined
Intervention/exposure	Ultrasound-guided peripheral cannulation with a specified target-vein variable: anatomical site or named vein, sonographic vessel depth, sonographic vessel diameter, or catheter-to-vein geometry	Landmark, near-infrared or transillumination guidance; studies in which the only variable was the catheter device or its length; operator-training interventions
Comparator	An alternative vein-selection strategy, an alternative depth, diameter or catheter-in-vein stratum, or a multivariable contrast within the same cohort; for single-arm series of a pre-specified target vein, an external reference (contemporaneous standard practice), which is not a concurrent comparison group	Ultrasound versus landmark comparisons with no stratification by target vein; no internal or external comparison
Outcomes	Primary: first-attempt and overall cannulation success. Secondary: catheter survival or dwell time, number of attempts, procedure time, complications	Studies reporting only satisfaction, cost, operator confidence or training metrics
Study design	Randomised controlled trials, prospective or retrospective cohort studies, prospective observational studies, secondary analyses of primary trial data	Reviews, meta-analyses, conference abstracts, editorials, letters, case reports, protocols and pre-prints
Other	Human studies in English in a peer-reviewed journal, database inception to 30 June 2026	Jugular (internal or external) cannulation; central venous, midline and peripherally inserted central catheters

Study Selection

Records were exported to a reference manager and de-duplicated electronically, followed by manual verification. Two reviewers independently screened titles and abstracts against the eligibility criteria, and the full texts of all potentially eligible records were retrieved and independently assessed. Disagreements at either stage were resolved by discussion, with recourse to a third reviewer where consensus could not be reached. Inter-reviewer agreement at the full-text stage was substantial (Cohen's κ = 0.82).

Data Extraction

Data were extracted independently and in duplicate onto a pre-piloted standardised form. Extracted fields comprised the first author, year of publication, country, study design, single- or multi-centre status, recruitment period, setting, definition of difficult access, sample size, participant demographics, operator grade and training, catheter gauge and length, the vein-selection variable and how it was measured, the comparator strategy or stratum, all reported outcomes with the corresponding effect estimates and measures of precision, and complication data. Where an article reported both crude and adjusted estimates, the adjusted estimate from the fully specified multivariable model was extracted. Discrepancies between extractors were reconciled against the source article.

Risk-of-Bias Assessment

All included studies were non-randomised studies of an intervention or exposure, so risk of bias was assessed using the Risk Of Bias In Non-randomised Studies of Interventions (ROBINS-I) tool across its seven domains: confounding, selection of participants, classification of interventions, deviations from intended interventions, missing data, measurement of outcomes, and selection of the reported result [9]. ROBINS-I was applied uniformly because every included study was non-randomised and the review question concerns a choice the operator makes, so each study could be assessed against a hypothetical target trial comparing one vein-selection strategy with another. In most included studies the "intervention" was not assigned but was a vein characteristic (depth, diameter, site or intraluminal catheter length) measured at the time of cannulation; these were treated as exposure contrasts within the same cohort (for example, deeper versus shallower veins, or upper-arm versus forearm sites), with the alternative stratum serving as the comparator. The domains were operationalised as follows. Confounding addressed patient and operator factors that could influence both the vein chosen and the outcome, principally body habitus, severity of difficult access, operator experience and catheter type; a low rating required either multivariable adjustment for such factors or a target fixed by protocol rather than chosen in response to patient characteristics. Selection of participants addressed whether inclusion depended on the exposure or on early outcomes, such as exclusion of failed attempts or of records lacking sonographic measurements. Classification of interventions addressed whether the vein characteristic was measured sonographically at the time of the procedure rather than inferred from records. Deviations from intended interventions addressed co-interventions that differed between strata, chiefly catheter length and gauge, and switching to another vein after a failed attempt. Missing data, measurement of outcomes and selection of the reported result were applied as specified in the tool [9]. For the two single-arm series that evaluated a pre-specified deep brachial or basilic strategy without an internal control group [10,11], the comparator was defined a priori as contemporaneous standard practice, that is, ultrasound-guided or landmark cannulation of conventional distal targets with standard-length catheters as reported in the emergency medicine literature of the same period. This external reference was used only to frame the risk-of-bias judgements; it is not a concurrent comparison group, and no comparative effect estimate was derived from it. Study-specific justifications for each domain-level judgement are provided in Appendix 2. Two reviewers assessed each study independently and assigned domain-level and overall judgements of low, moderate, serious or critical risk, with disagreements resolved by consensus.

Data Synthesis

The included studies differed substantially in design, in how vein-selection variables were categorised and in how outcomes were defined and censored, so statistical pooling would have produced an estimate without a coherent clinical interpretation. Synthesis was therefore narrative and structured by vein-selection domain: sonographic vessel diameter, sonographic vessel depth, anatomical site and named vein, and catheter-to-vein geometry. Within each domain, effect estimates were tabulated with their confidence intervals, the direction and consistency of the signal were assessed, and discordant findings were interrogated against study design and risk of bias. Descriptive statistics reported in the source articles were reproduced without recalculation.

Results

Study Selection

The searches returned 217 records: 68 from PubMed/MEDLINE, 73 from Embase, 42 from Scopus and 34 from Web of Science. After 114 duplicates were removed, 103 records were screened by title and abstract and 42 were excluded. All 61 reports sought for retrieval were obtained, and 51 were excluded at full-text assessment because no vein-selection variable was reported (n = 15), because the population was not emergency-department based (n = 17), or because the report was a non-original publication or lacked any comparison of vein-selection strategies (n = 19). Ten studies met all eligibility criteria and were included in the qualitative synthesis (Figure 1). Because 10 or fewer studies were eligible, no date restriction was applied and the evidence base is presented in full from 1999 to 2026.

**Figure 1:**
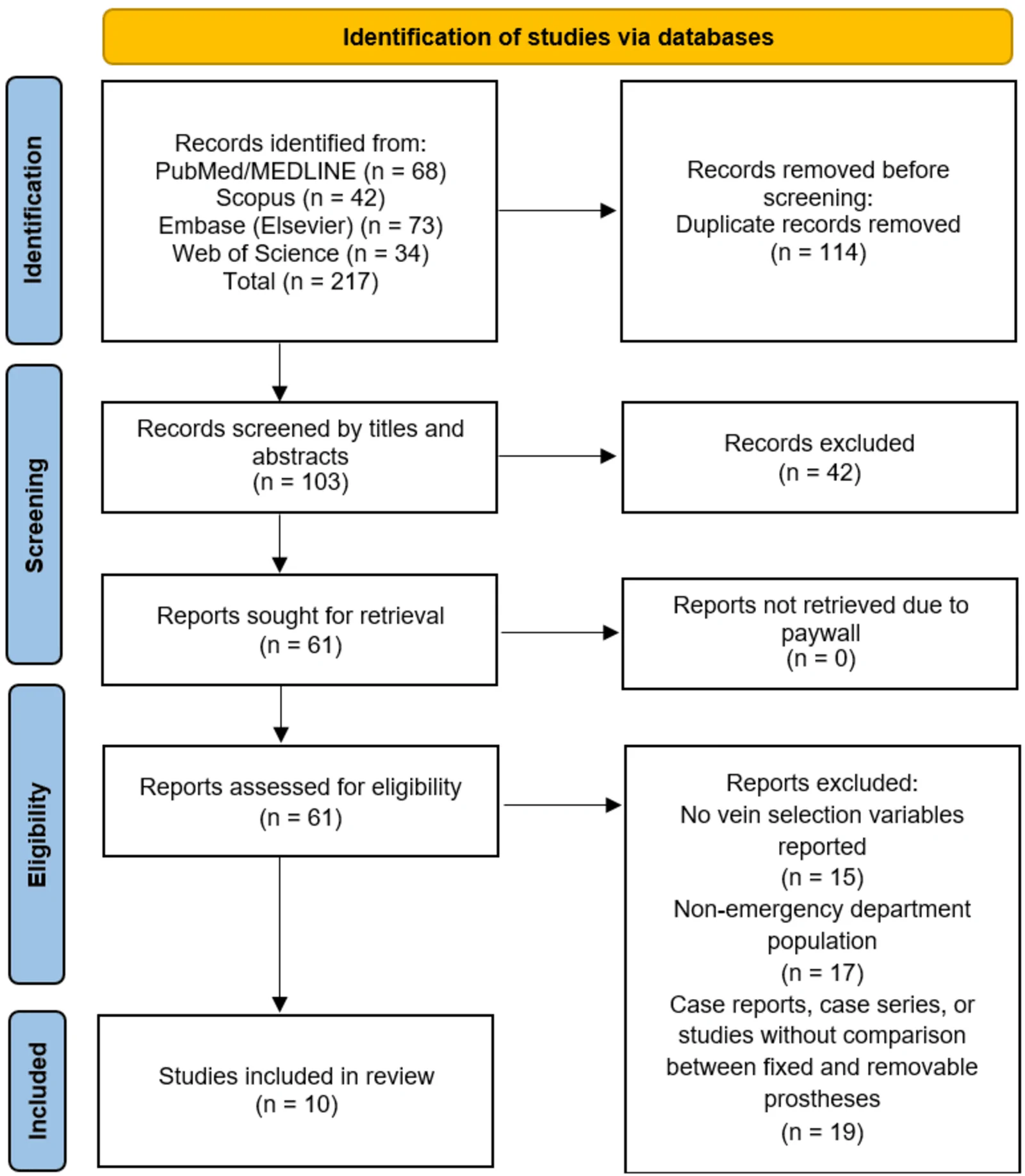
PRISMA 2020 flow diagram of study identification, screening and inclusion PRISMA: Preferred Reporting Items for Systematic Reviews and Meta-Analyses

Study Characteristics

All 10 studies were conducted in the United States and all were single-centre or two-centre investigations. Designs comprised four prospective observational cohorts [10-13], a secondary analysis of a prospectively gathered cohort with retrospective outcome follow-up [14], a prospective observational study with structured inpatient follow-up [15], a non-randomised prospective observational study spanning emergency and critical care placements [16], a retrospective analysis of a departmental quality-assurance database [17], a secondary analysis of prospectively collected randomised trial data [18] and a further retrospective quality-assurance cohort [19]. Together the studies contributed 1,952 patients or cannulation encounters, with individual sample sizes ranging from 25 [11] to 498 [19]. Difficult access was defined by two or more failed landmark attempts in every study, with three studies additionally accepting absent visible or palpable veins or a documented history of difficult access [12,17,19]. Catheters were placed by emergency physicians and residents in the earlier studies [10-14] and predominantly by trained emergency nurses in the more recent cohorts [16,17]. Full characteristics are presented in Table 2.

**Table 2:** Characteristics of the included studies

Author	Year	Country	Study design	Population	n	Exposure (vein-selection strategy)	Comparator	Outcomes	Key findings
Keyes et al. [10]	1999	USA	Prospective cohort	ED adults after ≥2 failed attempts; 50% injection drug use	101	Deep brachial or basilic vein; 5-cm catheter	Superficial distal veins (usual practice)	Success, time, early loss, complications	91% overall and 73% first-attempt success; 8% lost within 1 h; 2% arterial puncture
Mills et al. [11]	2007	USA	Prospective cohort	ED adults after two failed attempts	25	Deep brachial or basilic vein with wire-exchanged 15-cm catheter	Standard 3–5 cm catheters in the same veins	Success, access retained to 3 days, complications	92% success; all catheters functional to three days; 4% early infiltration
Panebianco et al. [12]	2009	USA	Prospective observational	Difficult-access patients in an urban tertiary ED	169 (236 attempts)	Target-vein diameter and depth	Diameter and depth strata	Successful placement	Success rose with diameter (OR 1.79 per 0.1 cm); no vein cannulated beyond 1.6 cm depth
Witting et al. [13]	2010	USA	Prospective observational	Cannulations from antecubital fossa to mid-humerus; 35 operators	180 encounters	Vein width and depth	Width and depth strata	First-puncture and eventual success	56% first-puncture and 84% eventual success; better at ≥0.4 cm width and 0.3–1.5 cm depth
Fields et al. [14]	2012	USA	Secondary analysis with retrospective follow-up	Difficult-access ED patients; uniform 20 G, 48-mm catheter	151	Vessel depth, diameter and anatomical site	Depth, diameter and site strata	48-hour catheter survival	Survival fell from 1.00 to 0.29 across depth strata; 0.83 forearm/antecubital versus 0.50 brachial
Pandurangadu et al. [15]	2018	USA	Prospective observational	ED adults followed as inpatients	86 (113 enrolled)	Proportion of catheter lying intraluminally	<30%, 30–64% and ≥65% strata	Functionality to 72 h	All failed at <30%; none failed at ≥65%; HR 0.71 per 5% increase
Miles et al. [16]	2021	USA	Prospective observational	Admitted difficult-access adults; ED or intensive care placement	97 (98 enrolled)	In-vein catheter length	Length as a continuous predictor	Catheter survival time	Mean survival 2.39 days; no association with in-vein length (p = 0.86)
Malik et al. [17]	2023	USA	Retrospective cohort	Nurse-placed catheters after two failed palpation attempts	388 (340 admitted)	Insertion site: forearm, antecubital fossa or upper arm	Upper arm versus antecubital and forearm	Dwell time, survival, failure	Median dwell 40.3 h; 6.2% failure; shorter dwell with upper-arm site in obese patients
Bahl et al. [18]	2024	USA	Secondary analysis of trial data	ED adults with difficult access requiring admission	257	Vein depth, extremity and intraluminal catheter length	Multivariable contrasts	Survival to completion of therapy	63% survived; depth >1.2 cm and right extremity raised failure; >2.75 cm in vein protective
Patten et al. [19]	2026	USA	Retrospective cohort	ED adults receiving ultrasound-guided catheters, 2020–2022	498	Vessel depth at the selected target	Depth as a continuous predictor	Survival to discharge or ≥72 h	66.5% survived; each additional millimetre of depth lowered survival (OR 0.91)

Synthesis by Vein-Selection Domain

Findings were grouped into four vein-selection domains, and the key estimates from each are summarised in Table 3.

**Table 3:** Summary of main findings by vein-selection domain OR: odds ratio; aHR: adjusted hazard ratio. Odds ratios and relative success refer to insertion success unless stated otherwise; hazard ratios refer to catheter failure. The composite heuristic row is a hypothesis-generating summary of thresholds reported in separate studies, not the result of any single analysis.

Vein-selection domain	Studies	Key estimates	Interpretation
Vessel diameter	[12-14]	OR 1.79 per 0.1 cm for insertion success [12]; 63% versus 39% first-puncture success at ≥0.4 cm (relative success 1.6) [13]; no statistically significant association with 48-hour survival [14]	An insertion-phase determinant only; threshold near 0.4 cm
Vessel depth	[12-14,18,19] (insertion: [12,13]; survival: [14,18,19])	No vein cannulated beyond 1.6 cm [12]; 58% versus 27% success within 0.3–1.5 cm [13]; survival 1.00 to 0.29 across depth strata [14]; aHR 1.68 for failure above 1.2 cm [18]; OR 0.91 per mm for survival [19]	The most reproducible signal; shallower targets were associated with better catheter survival in all three survival studies, with a threshold near 1.2 cm; insertion ceiling near 1.5–1.6 cm in two studies
Anatomical site and named vein	[10,11,14,17,18]	91% success with deep brachial or basilic targets [10,11]; 48-hour survival 0.83 forearm/antecubital versus 0.50 brachial [14]; shorter dwell with upper-arm sites in obesity [17]; aHR 1.64 for the right extremity [18]	Proximal deep veins maximise obtaining access; distal veins maximise retaining it
Catheter-to-vein geometry	[11,15,16,18]	100% failure below 30% intraluminal catheter and none at ≥65% [15]; aHR 0.58 for failure above 2.75 cm in vein [18]; no statistically significant association in one cohort [16]	Three of four studies support a threshold effect; the discordant cohort had unusually high baseline survival
Composite heuristic (not a validated decision rule)	[14,15,17-19]	Recurring optimum: diameter ≥0.4 cm, depth ≤1.2–1.5 cm, site at or distal to the antecubital fossa, ≥65% of catheter intraluminal; each threshold comes from a different cohort	Derived from concordant observational data; never tested prospectively as an intervention. The individual thresholds originate from separate cohorts with different populations, devices and outcome definitions; the combined rule has never been evaluated in a single population and should not be used as a validated clinical decision rule

Sonographic Vessel Diameter

Three studies related target-vessel calibre to outcome. In a prospective study of 169 DIVA patients undergoing 236 cannulation attempts, each 0.1-cm increment in vessel diameter raised the odds of successful placement by nearly 80% (OR 1.79, 95% CI 1.37-2.34), and the association was monotonic across the observed range [12]. A parallel prospective series of 180 cannulation encounters performed by 35 operators between the antecubital fossa and mid-humerus found first-skin-puncture success of 63% in veins of at least 0.4 cm compared with 39% in narrower vessels, a relative success of 1.6 (95% CI 1.1-2.3; p = 0.005) [13]. In the only study that examined longevity, a cohort of 151 DIVA patients all receiving an identical 20-gauge, 48-mm catheter, no statistically significant association between vessel diameter and 48-hour survival was detected [14]; a non-significant result in a single cohort does not establish that diameter has no effect on survival. On current evidence, diameter is therefore established only as an insertion-phase determinant, and the practical threshold reproduced across both insertion studies lies at approximately 0.4 cm.

Sonographic Vessel Depth

Depth was the most frequently examined and the most consistently informative variable, appearing in five studies, two of which assessed insertion success [12,13] and three catheter survival [14,18,19]. Its relationship with insertion success is non-linear. Increasing depth did not reduce the odds of successful cannulation in a continuous model (OR 0.96 per 0.1 cm, 95% CI 0.89-1.04), but beyond a threshold of 1.6 cm no vessel was successfully cannulated in 236 attempts [12]. A second study located an optimal window rather than a simple ceiling, reporting first-puncture success of 58% for veins at 0.3-1.5 cm compared with 27% for veins either shallower or deeper than that window (relative success 2.2, 95% CI 0.9-5.1; p = 0.04), the loss at the shallow extreme reflecting an oblique needle trajectory and a short intraluminal course [13].

In the three studies that examined catheter survival, the gradient was steeper and consistent. Forty-eight-hour survival probability fell from 1.00 in vessels less than 0.4 cm deep, to 0.62 at 0.40-1.19 cm, to 0.29 at 1.2 cm or more (p < 0.0001), and the effect became statistically apparent within three hours of insertion [14]. Multivariable analysis of 257 prospectively enrolled DIVA patients reproduced the same threshold, with vein depth greater than 1.2 cm carrying an adjusted hazard ratio for failure of 1.68 (95% CI 1.06-2.66; p = 0.03) [18]. The most recent and largest cohort, comprising 498 ultrasound-guided catheters, confirmed the association on a continuous scale: mean depth was 6.4 mm among catheters surviving to discharge or beyond 72 hours versus 7.5 mm among those that failed (OR for survival 0.91 per additional millimetre, 95% CI 0.86-0.96) [19]. The two insertion studies therefore identify an upper depth limit for successful cannulation, while all three survival studies, published between 2012 and 2026, consistently associate greater depth with catheter failure. The direction and precision of the individual, unpooled effect estimates across the vein-selection domains, together with the depth-stratified survival gradient, are displayed in Figure 2.

**Figure 2:**
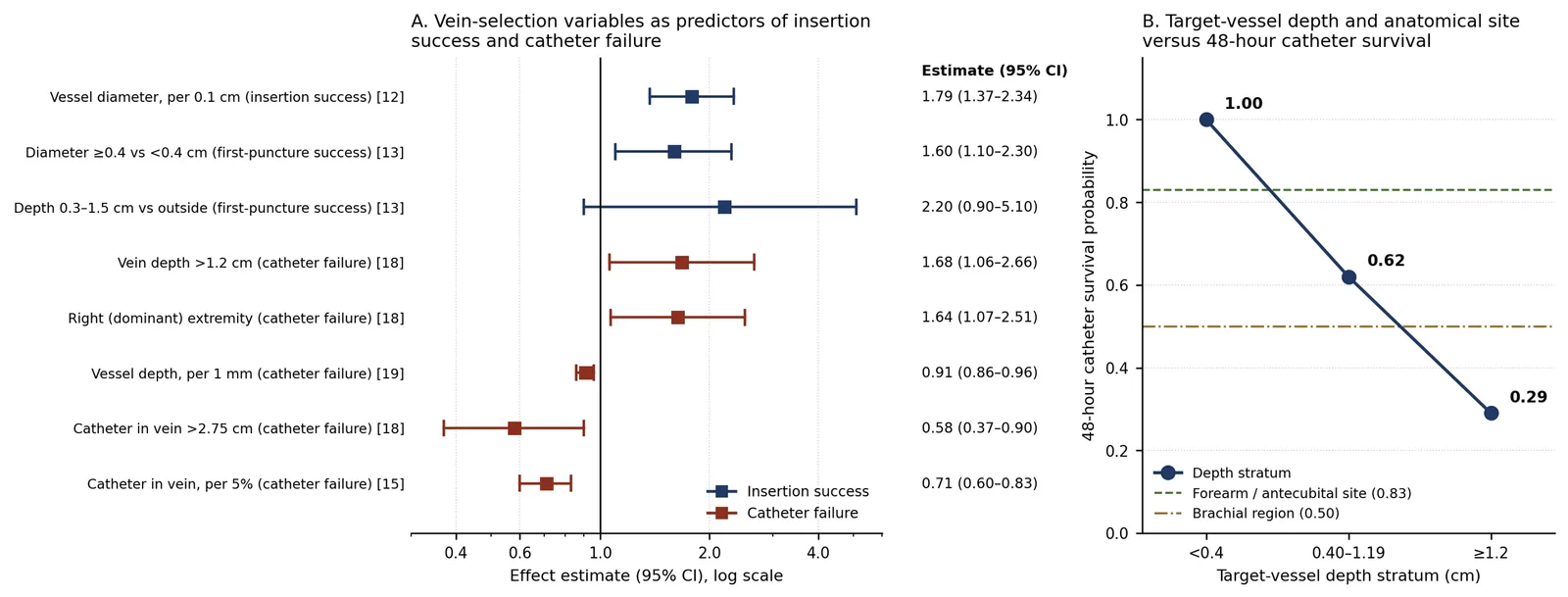
Effect estimates for vein-selection variables and stratified 48-hour catheter survival Panel A shows point estimates with 95% confidence intervals for vein-selection variables as predictors of insertion success (odds ratios and relative success) and of catheter failure (hazard and odds ratios), plotted on a logarithmic scale. Estimates are displayed individually and were not pooled. Odds ratios, relative success and hazard ratios are different measures and are not directly comparable: estimates for [12] and [13] refer to insertion success, hazard ratios for [15] and [18] refer to catheter failure, and the odds ratio for [19] refers to catheter survival per additional millimetre of depth. Reference numbers cited within the figure correspond to the following studies: [12], [13], [15], [18], and [19]. Panel B shows 48-hour Kaplan-Meier catheter survival probability by target-vessel depth stratum, with the corresponding site-specific survival probabilities as reference lines. CI: confidence interval

Anatomical Site and Named Vein

Five studies addressed site selection. The strategy of deliberately abandoning the depleted distal circulation in favour of the deep brachial or basilic vein was first evaluated prospectively in 101 ED patients with two prior failed attempts, half of whom were injection drug users; overall success was 91%, first-attempt success 73% and mean cannulation time 77 seconds, at the cost of brachial artery puncture in 2% and early loss of access in 8% within one hour [10]. Recognising that early dislodgement was the principal weakness of this approach, a subsequent prospective cohort combined the same deep targets with a wire-exchanged 15-cm catheter in 25 patients, achieving 92% success (95% CI 75-98%) catheters were left in place for up to three days by protocol; the median duration of access was 26 hours (interquartile range 10-47 hours), and the only reported complication was a single early infiltration [11].

The durability penalty attached to proximal targets was quantified directly in the 151-patient longevity cohort, in which 48-hour survival probability was 0.83 for catheters sited in the forearm or antecubital fossa but only 0.50 in the brachial region (p = 0.0002), with the location effect emerging after approximately 18 hours [14]. A retrospective analysis of 388 nurse-placed catheters found a compatible pattern confined to the subgroup in whom it would be expected to matter most: among obese patients, upper-arm placement conferred a shorter mean dwell time than antecubital and forearm placement considered together (38.6 versus 44.6 hours; p = 0.03), although no site effect on survival probability was demonstrable in the cohort as a whole, in which overall failure was low at 6.2% [17]. Laterality also proved relevant, with placement in the right (predominantly dominant) extremity independently associated with failure (adjusted hazard ratio 1.64, 95% CI 1.07-2.51; p = 0.02) [18]. Taken together, these data describe a dissociation rather than a contradiction: proximal deep veins maximise the probability of obtaining access, whereas distal veins maximise the probability of retaining it.

Catheter-to-Vein Geometry

Four studies examined the geometric consequence of vein choice, namely how much of the catheter finishes inside the vessel. Post-placement sonographic measurement in 86 ED patients followed as inpatients produced the sharpest threshold in the review: every catheter failed when less than 30% of its length lay intraluminally, 32.4% failed at 30-64%, and none failed when at least 65% was intraluminal (p < 0.0002), with each 5% increment reducing the hazard of failure by 29% (HR 0.71, 95% CI 0.60-0.83) [15]. Multivariable analysis of 257 patients converted the same relationship into an absolute target, with more than 2.75 cm of catheter in the vein independently protective (adjusted hazard ratio for failure 0.58, 95% CI 0.37-0.90; p = 0.01) [18]. The earlier deep-vein series is concordant in principle, since deliberately pairing a deep target with a 15-cm catheter was followed by only a single early infiltration [11], although without a concurrent comparison group it cannot show that early dislodgement was prevented. One study dissented: in 97 admitted patients whose catheters were placed in the ED or the intensive care unit, survival averaged 3,445 minutes (2.39 days) and showed no statistically significant association with in-vein catheter length (χ² = 0.03, p = 0.86), an outcome the authors attributed to improved operator proficiency and updated equipment rather than to geometry [16].

Clinical Outcomes and Complications

Outcome reporting was markedly heterogeneous, as summarised in Table 4. Only four studies reported first-attempt success, which ranged from 56% [13] to 73% [10], while overall success ranged from 84% [13] to 92% [11]. Catheter survival metrics were mutually incompatible: 48-hour survival probability [14], median time to failure among failures [15], mean survival in minutes [16], median dwell time with 96-hour survival probability [17], survival to completion of therapy [18] and survival to discharge or 72 hours [19] were each used once. Premature failure varied more than five-fold, from 6.2% [17] to 37% [18], a spread that tracks the censoring convention at least as closely as it tracks clinical performance. Complications were reported systematically in only the two earliest studies, which recorded brachial artery puncture in 2% and severe pain in 1% [10] and early infiltration in 4% with no thrombotic or infectious events [11]; no study identified a catheter-related bloodstream infection or symptomatic thrombosis attributable to a particular vein-selection strategy. This should not be read as evidence that any vein-selection strategy is free of these complications. Eight of the 10 studies did not assess complications systematically, follow-up in most studies was too short or too incomplete to capture delayed events such as thrombosis or bloodstream infection, and no study was designed or powered to compare complication rates between vein-selection strategies; the absence of reported events therefore reflects an absence of evidence rather than evidence of absence.

**Table 4:** Clinical and catheter-related outcomes reported by the included studies NR: Not reported; ED: Emergency department; IQR: interquartile range. "NR" for complications indicates that complications were not systematically reported, not that none occurred.

Study	First-attempt success	Overall success	Catheter survival	Premature failure	Complications
Keyes et al. [10]	73%	91%	Not followed beyond the ED	8% within 1 h	Arterial puncture 2%; severe pain 1%
Mills et al. [11]	NR	92%	Median duration of access 26 h (IQR 10–47 h); left in place ≤3 days by protocol	One early infiltration	Early infiltration 4%
Panebianco et al. [12]	NR	Modelled per attempt	Not assessed	Not assessed	NR
Witting et al. [13]	56%	84%	Not assessed	Not assessed	NR
Fields et al. [14]	NR	NR	0.63 at 48 h	32% by 48 h	NR
Pandurangadu et al. [15]	NR	NR	Median 15.6 h to failure	33.7% by 72 h	NR
Miles et al. [16]	NR	NR	Mean 2.39 days	29.9%	NR
Malik et al. [17]	NR	83.9% usable at 1 h	Median dwell 40.3 h	6.2%	NR
Bahl et al. [18]	Modelled as a covariate	NR	63% to completion of therapy	37%	NR
Patten et al. [19]	NR	NR	66.5% to discharge or ≥72 h	33.5%	NR

Risk of Bias

Overall risk of bias was low in four studies [10,12,15,18], moderate in two [16,19] and serious in four [11,13,14,17]; no study was rated at critical risk (Figure 3). Classification of the exposure was at low risk in all 10 studies, because vein depth, diameter, site and intraluminal catheter length were measured sonographically at the time of the procedure rather than inferred from records. Confounding was the domain that most often limited confidence, being serious in the two small single-arm series in which no adjustment for patient or operator factors was possible [11,13] and moderate in four further cohorts in which target-vein choice remained at the operator's discretion [14,16,17,19]. Missing data were judged serious in the two studies in which a substantial proportion of eligible records could not be analysed [14,17], and moderate where follow-up documentation was incomplete [13,16,19]. Measurement of outcomes and selection of the reported result were at low or moderate risk throughout, the moderate ratings reflecting unblinded outcome ascertainment and, for the secondary analyses, the absence of a pre-specified analysis plan. The four studies at low overall risk comprised two prospective cohorts with complete protocol-driven follow-up [10,15], a prospective observational study with pre-specified sonographic measurements [12], and a secondary analysis of prospectively collected randomised trial data with full outcome ascertainment [18]. Study-specific justifications for every domain-level judgement, including the four studies rated at low risk of confounding [10,12,15,18], are given in Appendix 2.

**Figure 3:**
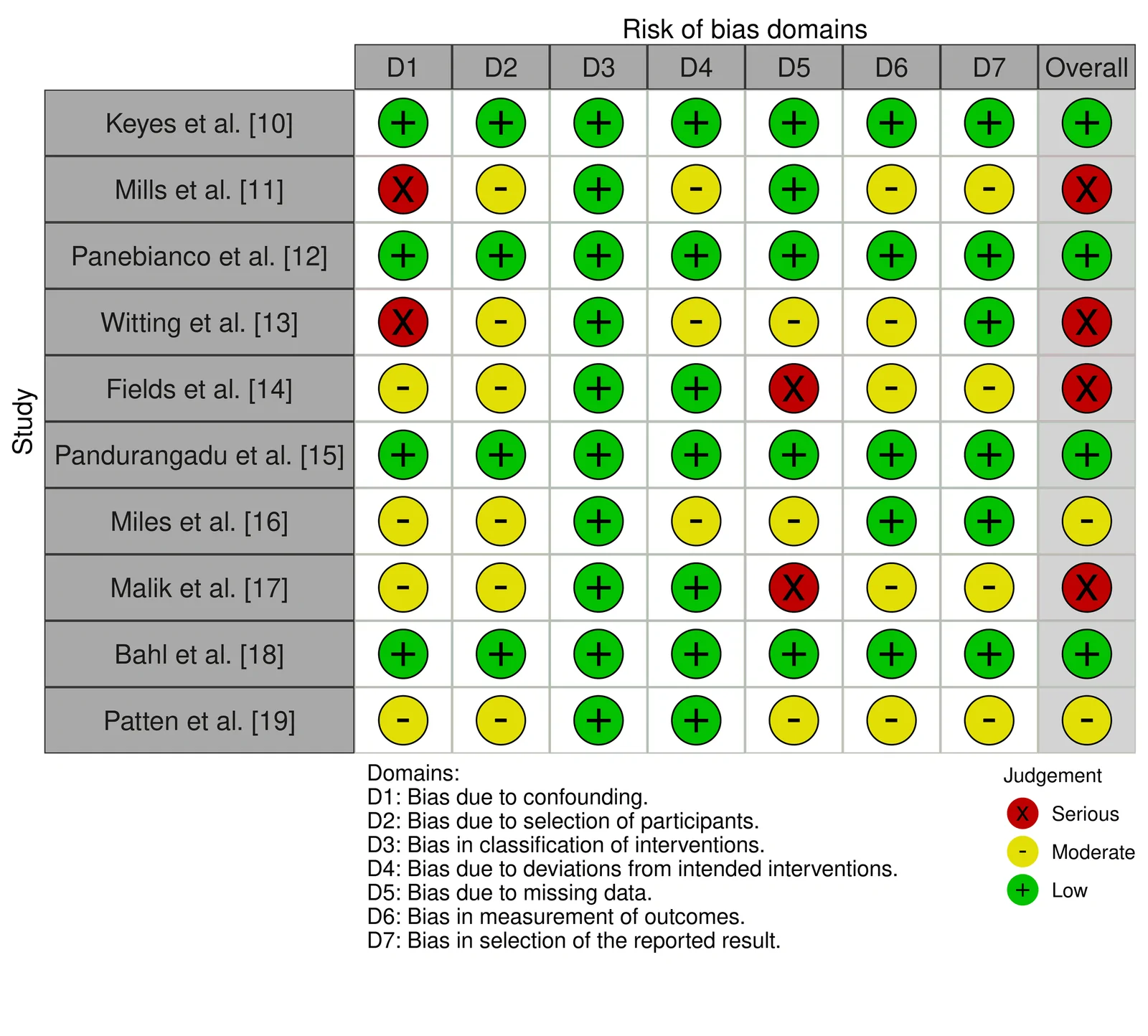
ROBINS-I traffic-light plot of domain-level and overall risk-of-bias judgements ROBINS-I: Risk Of Bias In Non-randomised Studies of Interventions. D1: bias due to confounding; D2: bias due to selection of participants; D3: bias in classification of interventions; D4: bias due to deviations from intended interventions; D5: bias due to missing data; D6: bias in measurement of outcomes; D7: bias in selection of the reported result. The reference numbers cited alongside the author names correspond to the following studies: Keyes et al. [10], Mills et al. [11], Panebianco et al. [12], Witting et al. [13], Fields et al. [14], Pandurangadu et al. [15], Miles et al. [16], Malik et al. [17], Bahl et al. [18], and Patten et al. [19].

Discussion

This review assembles, for the first time, the primary evidence bearing on a decision that ultrasound alone makes possible: which vein to cannulate in an ED patient whose superficial circulation has already failed. Ten studies contributing 1,952 patients or cannulation encounters converge on a finding that is simple to state and rarely acted upon in practice. The vein that most reliably yields access is frequently not the vein that most reliably retains it. Larger vessels are easier to cannulate, deeper vessels are harder to keep, proximal deep veins deliver the highest first-pass success and the poorest durability, and the amount of catheter that ends up inside the lumen - itself a direct arithmetic consequence of how deep and how large the chosen vein is - emerges repeatedly as the mechanical hinge on which longevity turns. Vein selection, in other words, is not a preliminary to the procedure; it is the procedure's most consequential and most modifiable step.

The evidence for target-vessel calibre is internally coherent and confined to the insertion phase. Both insertion studies found that success rose with diameter [12,13]. The mechanism is unremarkable: a wider lumen tolerates greater angular error in needle trajectory and offers a larger target in the transverse plane. No statistically significant association with catheter survival was detected in the one study that examined it [14]; a single non-significant result cannot exclude a modest effect, but it is plausible that failure after insertion is driven more by mechanical dislodgement, positional occlusion and infiltration than by lumen size. Diameter should therefore be read as a criterion for choosing among candidate veins when the immediate objective is to obtain access rapidly in an unstable patient, and not as a reassurance about the line's future.

Depth behaves differently, and its behaviour is the most robust finding in this review. The relationship with insertion success is not linear but bounded: within an intermediate window the operator is largely indifferent to depth, but a ceiling exists beyond which cannulation with standard-length devices simply does not succeed, located at 1.6 cm in one cohort [12] and reflected in the 0.3-1.5 cm optimal window identified in another [13]. The relationship with catheter survival, by contrast, is a continuous and steep gradient, observed in all three studies that examined it [14,18,19]. That the two insertion studies and the three survival studies, published between 2009 and 2026 in different institutions and using different catheter gauges and lengths, should point in the same direction is unusual in this literature and lends the depth threshold a practical authority that few other findings here possess. The pattern is consistent with the geometric mechanism outlined in the Introduction.

That mechanism is precisely what the catheter-to-vein geometry studies measured directly, and their findings therefore corroborate rather than merely accompany the depth data. The sharp intraluminal-proportion threshold [15] and the absolute target of more than 2.75 cm of intravascular catheter [18] give the clinician a number that can be verified at the bedside immediately after insertion. The single discordant study is instructive rather than merely inconvenient. Its non-significant result arose in a cohort with unusually high overall survival [16], and a null finding in 97 patients cannot exclude an effect. The most reasonable reading is that geometric optimisation matters most when baseline performance is poor, and that a highly proficient service using appropriate catheters may already sit above the threshold in most insertions, leaving little residual variance for in-vein length to explain.

The site data resolve an apparent contradiction that has long confused bedside teaching. The deep brachial and basilic veins were the original ultrasound targets precisely because they remain patent when the distal circulation is exhausted, and they yielded the highest insertion success in this review [10,11]. Yet the same anatomical choice underlies the poorest durability observed in this review [14,17]. Rather than a conflict, this is a coherent trade-off between two different clinical objectives, and one potential resolution was suggested as early as 2007, when a deep target paired with a 15-cm wire-exchanged catheter retained high insertion success with only a single early infiltration [11]; however, the median duration of access in that series was 26 hours and, without a comparison group, it cannot show that dislodgement was prevented. Vein selection and device selection are therefore not independent decisions, and any selection rule that specifies a target vein without specifying a matched catheter length is incomplete.

These findings sit comfortably alongside the wider literature excluded from this review by design. The studies discussed in this paragraph did not meet the eligibility criteria and contribute nothing to the synthesis above; they are cited only as context. The peripheral internal jugular or “easy IJ” technique represents the logical extension of the same escalation logic - when the arm is exhausted, move to a larger and more reliable vein - and prospective ED series report insertion success of 88% with a mean procedure time of 4.4 minutes and loss of patency in 14% [20], and 97.1% success with a single local haematoma among 35 patients [21]. Although these series cannot be compared directly with the upper-limb studies, they suggest that the vein-selection gradient described here does not stop at the shoulder, and that the neck is being used, informally and without an evidence-based selection rule, as the final rung of the same ladder. Device-focused studies point in the same direction from a different angle: longer catheters outperform standard-length devices in ultrasound-guided cannulation [22], and a randomised comparison of ultralong against standard long catheters found survival differences attributable to the length of catheter available to traverse subcutaneous tissue [23]. Read together with the present synthesis, these literature studies describe one underlying variable - the intravascular fraction of the catheter - approached from opposite ends by investigators who chose to vary either the vein or the device.

Pending prospective validation, these findings support an evidence-informed approach rather than a definitive clinical protocol. For the stable DIVA patient who requires access for more than a few hours, a reasonable approach suggested by the observational data is to select a vein of at least 0.4 cm in diameter, no deeper than approximately 1.2 cm, at or distal to the antecubital fossa, in the non-dominant limb, and to confirm sonographically after insertion that most of the catheter lies intraluminally, choosing a longer device if the chosen depth makes that impossible. These parameters are drawn from separate cohorts with different populations, devices and outcome definitions; they have never been evaluated together in a single population and should not be interpreted as a validated clinical decision rule. When immediate access outweighs durability, the deep brachial or basilic vein offered the highest insertion success in the included studies and may be considered, with the expectation that the line may need early replacement and with attention to the risk of brachial artery puncture [10]; this rests on two small single-centre series [10,11] and has not been compared directly with other targets. Framing the decision explicitly as a trade-off between obtaining and retaining access, rather than as a search for a single best vein, may improve both bedside teaching and departmental protocols. It also has implications for how ultrasound-guided access is taught, since current curricula concentrate on needle-tip tracking and probe orientation while devoting comparatively little attention to the antecedent question of target selection.

Several knowledge gaps are conspicuous. First, no included study prospectively tested a composite selection rule as an intervention; every recommendation available to clinicians is derived from observational associations, several of them from secondary analyses of data collected for other purposes. A randomised comparison of protocolised sonographic vein selection against operator discretion, with first-attempt success and 72-hour survival as co-primary outcomes, is both feasible and overdue. Second, all ten studies were conducted in the United States and none used a directly comparable survival endpoint, which limits both generalisability and any future attempt at quantitative pooling; the field would benefit from a core outcome set specifying uniform censoring conventions, as the five-fold spread in reported failure rates in this review reflects methodological convention at least as much as clinical performance. This variability is well recognised, with survival in unselected DIVA cohorts reported as low as 56% with a median of 26 hours and 47% failure within the first day [24]. Third, obese patients, in whom depth is by definition greater and in whom the observed site effect was strongest [17], have never been studied as a pre-specified subgroup, despite being the population in which selection rules would matter most. Fourth, evidence from adjacent settings suggests the principles generalise - ultrasound-guided cannulation of deep arm veins with long peripheral catheters performed well in post-cardiac-surgery patients with difficult access [25] - but this has not been formally tested against ED cohorts. Finally, patient-centred outcomes were almost entirely absent: no included study reported pain, satisfaction or the number of subsequent venipunctures as a function of the vein selected, even though these are the outcomes DIVA patients themselves consistently prioritise.

The strengths of this review include a comprehensive multi-database search with hand-searching and citation tracking, duplicate screening and extraction, a pre-specified PICOS framework, and formal risk-of-bias assessment using ROBINS-I. Its principal limitation is the quality of the underlying evidence: no randomised trial has yet allocated patients to a vein-selection strategy, four of ten studies were at serious risk of bias, and confounding by indication is unavoidable in a literature where the operator chooses the target after assessing the patient. Consequently the associations reported here should be read as robust and reproducible signals for hypothesis-driven trial design rather than as established causal effects.

Limitations

Several limitations warrant explicit statement. The review was not prospectively registered, which increases the theoretical risk of post hoc changes to scope, although the protocol was written before the searches were run. Only English-language, peer-reviewed publications were eligible, raising the possibility of language and publication bias; relevant evidence published in other languages or outside peer-reviewed journals may therefore have been excluded, and small negative studies of vein selection may be particularly liable to remain unpublished. Formal assessment of publication bias by funnel plot was not feasible with ten heterogeneous studies. Because outcome definitions, censoring rules and follow-up horizons differed across every study, meta-analysis was not performed and no pooled effect estimate is offered; the synthesis is narrative and the thresholds reported should be regarded as convergent rather than statistically integrated. Complications were assessed systematically in only two studies, so this review cannot compare the safety of vein-selection strategies, and the absence of reported bloodstream infection or thrombosis should not be interpreted as evidence that these complications do not occur. All included studies originated in the United States and most were single-centre, so applicability to systems with different staffing models, catheter availability and admission pathways is uncertain. Jugular and central approaches, paediatric populations and purely non-emergency settings were excluded by design, so the conclusions apply specifically to upper-extremity peripheral access in adult ED patients. Finally, ROBINS-I is designed for comparative non-randomised studies, and its application to the single-arm cohorts included here required treating contemporaneous standard practice as an implicit comparator; the resulting judgements for those studies should be interpreted with corresponding caution.

## Conclusions

Across 10 primary studies in adult ED patients with difficult intravenous access, the choice of target vein was consistently associated with both the probability of successful ultrasound-guided cannulation and the durability of the resulting catheter, but not always in the same direction. Larger vessel diameter was associated with improved insertion success, whereas greater vessel depth was the most reproducible factor associated with poorer catheter survival and with a lower likelihood of successful insertion.

Deep proximal upper-arm veins offered the highest first-pass success but the shortest dwell times, while forearm and antecubital targets showed the opposite pattern, and this trade-off may be mitigated, on the evidence of one small uncontrolled series, when a deep target was paired with a catheter long enough to leave most of its length intraluminally. The available evidence is entirely observational, geographically homogeneous and at serious risk of bias in a substantial minority of studies, so these findings define a coherent and physiologically plausible selection heuristic rather than an established standard; because its component thresholds derive from separate cohorts, the heuristic should be regarded as an evidence-informed approach pending prospective validation, not as a validated clinical decision rule. A randomised comparison of protocolised sonographic vein selection against operator discretion, using an agreed core outcome set that includes patient-reported measures, is the necessary next step.

## Appendices

Appendix 1

**Table 5:** Search strategies for electronic databases

Database	Search strategy as executed	Limits/filters	Records retrieved
PubMed / MEDLINE (NLM interface)	#1 "Ultrasonography"[Mesh] OR "Ultrasonography, Interventional"[Mesh] OR ultrason*[tiab] OR ultrasound*[tiab] OR sonograph*[tiab] OR echograph*[tiab] OR "point of care ultrasound"[tiab] OR "point-of-care ultrasound"[tiab] OR POCUS[tiab]#2 "Catheterization, Peripheral"[Mesh] OR "Administration, Intravenous"[Mesh] OR "peripheral intravenous"[tiab] OR "peripheral venous"[tiab] OR "intravenous access"[tiab] OR "venous access"[tiab] OR "vascular access"[tiab] OR cannulat*[tiab] OR catheteri*[tiab] OR PIVC[tiab] OR PIV[tiab] OR USGPIV*[tiab] OR USPIV*[tiab]#3 "Emergency Service, Hospital"[Mesh] OR "Emergency Medicine"[Mesh] OR "emergency department"[tiab] OR "emergency departments"[tiab] OR "emergency service"[tiab] OR "emergency room"[tiab] OR "difficult intravenous access"[tiab] OR "difficult venous access"[tiab] OR "difficult vascular access"[tiab] OR "difficult IV access"[tiab] OR DIVA[tiab] OR "hard stick"[tiab]#4 "vein depth"[tiab] OR "vessel depth"[tiab] OR "venous depth"[tiab] OR "vein diameter"[tiab] OR "vessel diameter"[tiab] OR "vein width"[tiab] OR "vein size"[tiab] OR "vessel calib*"[tiab] OR "vein selection"[tiab] OR "target vein"[tiab] OR "insertion site"[tiab] OR "catheter to vein"[tiab] OR "catheter-to-vein"[tiab] OR "catheter in vein"[tiab] OR "catheter in-vein"[tiab] OR basilic[tiab] OR brachial[tiab] OR cephalic[tiab] OR forearm[tiab] OR antecubital[tiab] OR "upper arm"[tiab]#5 #1 AND #2 AND #3 AND #4#6 #5 AND English[lang]	English language	68
Embase (Elsevier, Embase.com)	#1 'echography'/exp OR 'ultrasound'/exp OR ultrason*:ti,ab,kw OR ultrasound*:ti,ab,kw OR sonograph*:ti,ab,kw OR echograph*:ti,ab,kw OR 'point of care ultrasound':ti,ab,kw OR pocus:ti,ab,kw#2 'peripheral catheterization'/exp OR 'intravenous catheter'/exp OR 'peripheral intravenous':ti,ab,kw OR 'peripheral venous':ti,ab,kw OR 'intravenous access':ti,ab,kw OR 'venous access':ti,ab,kw OR 'vascular access':ti,ab,kw OR cannulat*:ti,ab,kw OR catheteri*:ti,ab,kw OR pivc:ti,ab,kw OR usgpiv*:ti,ab,kw#3 'emergency ward'/exp OR 'emergency medicine'/exp OR 'emergency department':ti,ab,kw OR 'emergency room':ti,ab,kw OR 'difficult intravenous access':ti,ab,kw OR 'difficult venous access':ti,ab,kw OR 'difficult vascular access':ti,ab,kw OR diva:ti,ab,kw#4 'vein depth':ti,ab,kw OR 'vessel depth':ti,ab,kw OR 'vein diameter':ti,ab,kw OR 'vessel diameter':ti,ab,kw OR 'vein width':ti,ab,kw OR 'vein size':ti,ab,kw OR 'vein selection':ti,ab,kw OR 'target vein':ti,ab,kw OR 'insertion site':ti,ab,kw OR 'catheter to vein':ti,ab,kw OR 'catheter in vein':ti,ab,kw OR basilic:ti,ab,kw OR brachial:ti,ab,kw OR cephalic:ti,ab,kw OR forearm:ti,ab,kw OR antecubital:ti,ab,kw OR 'upper arm':ti,ab,kw#5 #1 AND #2 AND #3 AND #4#6 #5 AND [english]/lim AND [humans]/lim	English; humans	73
Scopus (Elsevier)	TITLE-ABS-KEY ( ultrason* OR ultrasound* OR sonograph* OR echograph* OR "point of care ultrasound" OR pocus )ANDTITLE-ABS-KEY ( "peripheral intravenous" OR "peripheral venous" OR "intravenous access" OR "venous access" OR "vascular access" OR cannulat* OR catheteri* OR pivc OR usgpiv* )ANDTITLE-ABS-KEY ( "emergency department" OR "emergency room" OR "emergency service" OR "difficult intravenous access" OR "difficult venous access" OR "difficult vascular access" OR diva )ANDTITLE-ABS-KEY ( "vein depth" OR "vessel depth" OR "vein diameter" OR "vessel diameter" OR "vein width" OR "vein size" OR "vein selection" OR "target vein" OR "insertion site" OR "catheter to vein" OR "catheter in vein" OR basilic OR brachial OR cephalic OR forearm OR antecubital OR "upper arm" )AND ( LIMIT-TO ( LANGUAGE , "English" ) )	English language	42
Web of Science (Clarivate Core Collection)	TS = ( ultrason* OR ultrasound* OR sonograph* OR echograph* OR "point of care ultrasound" OR POCUS )ANDTS = ( "peripheral intravenous" OR "peripheral venous" OR "intravenous access" OR "venous access" OR "vascular access" OR cannulat* OR catheteri* OR PIVC OR USGPIV* )ANDTS = ( "emergency department" OR "emergency room" OR "emergency service" OR "difficult intravenous access" OR "difficult venous access" OR "difficult vascular access" OR DIVA )ANDTS = ( "vein depth" OR "vessel depth" OR "vein diameter" OR "vessel diameter" OR "vein width" OR "vein size" OR "vein selection" OR "target vein" OR "insertion site" OR "catheter to vein" OR "catheter in vein" OR basilic OR brachial OR cephalic OR forearm OR antecubital OR "upper arm" )	English language	34

Appendix 2

**Table 6:** Study-specific justifications for ROBINS-I risk-of-bias judgements ROBINS-I: Risk Of Bias In Non-randomised Studies of Interventions

Study	Overall	Domain-level justification (D1 confounding; D2 selection; D3 classification; D4 deviations; D5 missing data; D6 outcome measurement; D7 reported result)
Keyes et al. [10]	Low	aD1 low: deep brachial or basilic target fixed by protocol for every enrolled patient, so the exposure was not chosen in response to patient characteristics, and the primary outcome (insertion success) was ascertained immediately in the ED. D2 low: prospective enrolment by a single protocol after two failed attempts. D3 low: target identified sonographically at the procedure. D4-D7 low: single standardised technique, complete ED follow-up, objective success definition, outcomes reported as specified. Comparator: contemporaneous standard practice (external reference, not a concurrent comparison group).
Mills et al. [11]	Serious	aD1 serious: single-arm series of 25 patients with no adjustment possible; the effect of the deep target on duration of access is inseparable from the wire-exchanged 15-cm device and from post-ED care. D2 moderate: small sample with limited description of eligible patients not enrolled. D3 low: target identified sonographically. D4 moderate: two-step device exchange as co-intervention. D5 low. D6 moderate: unblinded ascertainment of duration of access. D7 moderate: limited pre-specification. Comparator: contemporaneous standard practice (external reference).
Panebianco et al. [12]	Low	aD1 low: vein and patient characteristics analysed together in a multivariable model. D2 low: prospective enrolment of difficult-access patients irrespective of the vein chosen. D3 low: diameter and depth measured sonographically before each attempt. D4-D7 low: objectively defined per-attempt outcome, complete data, pre-specified measurements.
Witting et al. [13]	Serious	aD1 serious: unadjusted comparison of width and depth strata across 35 operators of differing experience. D2 moderate: encounter-level sampling. D3 low: sonographic measurement. D4 moderate: technique varied between operators. D5 moderate: incomplete follow-up documentation. D6 moderate: unblinded outcome ascertainment. D7 low.
Fields et al. [14]	Serious	aD1 moderate: depth and site chosen at operator discretion; a uniform 20-gauge, 48-mm catheter limited device confounding. D2 moderate. D3 low. D4 low: uniform device. D5 serious: a substantial proportion of eligible records could not be analysed. D6 moderate: retrospective outcome follow-up. D7 moderate: secondary analysis without a pre-specified plan.
Pandurangadu et al. [15]	Low	aD1 low: the exposure (proportion of catheter intraluminal) was measured by protocol after placement and analysed with time-to-event methods, limiting confounding by indication. D2 low: prospective enrolment. D3 low: post-placement sonographic measurement. D4-D7 low: protocol-driven inpatient follow-up with complete outcome ascertainment.
Miles et al. [16]	Moderate	aD1 moderate: vein chosen at operator discretion; ED and intensive care placements combined. D2 moderate. D3 low. D4 moderate: differing placement settings and equipment. D5 moderate: incomplete follow-up documentation. D6-D7 low.
Malik et al. [17]	Serious	aD1 moderate: insertion site chosen at operator discretion. D2 moderate. D3 low. D4 low. D5 serious: a substantial proportion of eligible records could not be analysed. D6 moderate: retrospective, unblinded outcome ascertainment. D7 moderate: quality-assurance database without a pre-specified analysis plan.
Bahl et al. [18]	Low	aD1 low: multivariable time-to-event model of prospectively collected trial data adjusting for patient and procedural covariates. D2-D7 low: protocol-driven data collection, sonographic measurements, full outcome ascertainment.
Patten et al. [19]	Moderate	aD1 moderate: target chosen at operator discretion. D2 moderate. D3 low. D4 low. D5 moderate: incomplete documentation. D6 moderate: unblinded retrospective ascertainment. D7 moderate: retrospective cohort without a pre-specified plan.
